# Synthesis and Characterization of Polyvinylpyrrolidone-Modified ZnO Quantum Dots and Their In Vitro Photodynamic Tumor Suppressive Action

**DOI:** 10.3390/ijms22158106

**Published:** 2021-07-28

**Authors:** Tianming Song, Yawei Qu, Zhe Ren, Shuang Yu, Mingjian Sun, Xiaoyu Yu, Xiaoyang Yu

**Affiliations:** 1 The Higher Educational Key Laboratory for Measuring & Control Technology and Instrumentations of Heilongjiang Province, Harbin University of Science and Technology, Harbin 150080, China; 1610600001@stu.hrbust.edu.cn (T.S.); 1910600001@stu.hrbust.edu.cn (Z.R.); yushuang@hrbust.edu.cn (S.Y.); 2 Department of Control Science and Engineering, Harbin Institute of Technology, Harbin 150000, China; 1910600002@stu.hrbust.edu.cn (Y.Q.); sunmingjian@hit.edu.cn (M.S.); 3 School of Atmospheric Sciences, Sun Yat-sen University, Guangzhou 519000, China

**Keywords:** zinc oxide, quantum dot, polyvinylpyrrolidone, photodynamic therapy, photosensitizers, reactive oxygen species, nanoparticle, SW480 cancer cell

## Abstract

Despite the numerous available treatments for cancer, many patients succumb to side effects and reoccurrence. Zinc oxide (ZnO) quantum dots (QDs) are inexpensive inorganic nanomaterials with potential applications in photodynamic therapy. To verify the photoluminescence of ZnO QDs and determine their inhibitory effect on tumors, we synthesized and characterized ZnO QDs modified with polyvinylpyrrolidone. The photoluminescent properties and reactive oxygen species levels of these ZnO/PVP QDs were also measured. Finally, in vitro and in vivo experiments were performed to test their photodynamic therapeutic effects in SW480 cancer cells and female nude mice. Our results indicate that the ZnO QDs had good photoluminescence and exerted an obvious inhibitory effect on SW480 tumor cells. These findings illustrate the potential applications of ZnO QDs in the fields of photoluminescence and photodynamic therapy.

## 1. Introduction

In recent years, cancer has become one of the most fatal diseases threatening human health and the second leading cause of mortality worldwide [1,2]. The current cancer treatments include surgery, radiotherapy, and chemotherapy. However, these therapies are associated with complications, such as tissue trauma, side effects, and reoccurrence.

Photodynamic therapy (PDT) is a cancer treatment modality that can be applied to treat various tumors. In PDT, light and photosensitizers (PS) are used to destroy tumor cells by generating reactive oxygen species (ROS) [3,4,5]. The basic principle of photodynamic therapy is that the use of a photosensitizer exposed to a certain wavelength of light will be excited to produce ROS, which can kill cancer cells [6]. Currently, the commonly used light source is ultraviolet (UV) light, and various new photosensitizers are under development, particularly nanomaterials [7], for use in research in the PDT field. Compared with other treatment modalities, PDT is noninvasive, has a lower incidence of trauma, and causes less toxicity and fewer side effects [8]. Currently, PDT has been approved for treating superficial tumors, including skin cancer [9], superficial bladder cancer [10], lung cancer [11], cervical cancer [12], and head and neck cancers [13,14].

Zinc oxide (ZnO), an inexpensive and versatile inorganic nanomaterial with photoelectric properties, is often used in photocatalysis and on ceramic surfaces. Recently, the synthesis and biomedical application of ZnO nanoparticles have gained attention [15,16,17,18,19,20,21,22,23,24,25] because of their anticancer, antibacterial, antioxidant, antidiabetic, and anti-inflammatory activities, as well as for drug delivery and bioimaging applications. ZnO NPs were also reported to induce cytotoxicity in a variety of cancer cells [26,27,28,29,30,31,32,33,34,35,36]. Zinc (Zn^2+^), a component of ZnO, is widely recognized as an essential micronutrient for humans and, thus, is safe for application. Furthermore, ZnO particles have been designated as Generally Recognized as Safe by the Food and Drug Administration [37].

ZnO quantum dots (QDs) are used for conducting PDT as a PS and for biological imaging. Gao et al. [38] combined ZnO with X-ray, MRI, and other imaging methods in vivo to monitor tumor growth and metastasis in real time. Fluorescence imaging, in contrast, exhibits the advantages of low cost, high sensitivity, and optimal spatial resolution among molecular imaging techniques. ZnO QDs possess excellent photodynamic properties and have potential utility in photoluminescence imaging. ZnO QDs are typically used in bioimaging [39,40] and drug delivery because of their high biocompatibility [41,42]. Many researchers aim to improve the stability and water solubility of ZnO QDs. PVP can serve as a surface stabilizer, growth modifier, nanoparticle dispersant, and reducing agent [43]. Several methods for synthesizing QDs have been reported, including coprecipitation, sol–gel, solid-state, and hydrothermal methods [44,45,46,47,48,49,50,51,52,53,54,55,56]. Each method has various advantages and limitations. For example, nanoparticles synthesized by the coprecipitation method are larger than those obtained by other methods, whereas the stability of the aqueous solution synthesized by the sol–sol method is lower than that observed by other methods. However, there are several limitations of ZnO QDs, including their poor water stability and easy agglomeration, preventing their application in the biological domain [57,58,59].

In this study, we developed an optimized method for synthesizing ZnO QDs modified with polyvinylpyrrolidone (PVP40) to improve their stability in aqueous solutions and investigated their characteristics and optical properties. Next, we assessed the cytotoxicity of ZnO/PVP QDs in SW480 cancer cells and HEK-293T human kidney cells to improve the biomedical applications of ZnO/PVP QDs. We evaluated the applications of ZnO/PVP QDs for both in vitro and in vivo PDT and analyzed the mechanisms and principles of PDT in cancer treatments. Our results indicate that ZnO QDs have considerable potential applications in the fields of photoluminescence and photodynamic tumor suppression.

## 2. Results and Discussion

### 2.1. Synthesis of ZnO/PVP QDs

**Figure 1:**
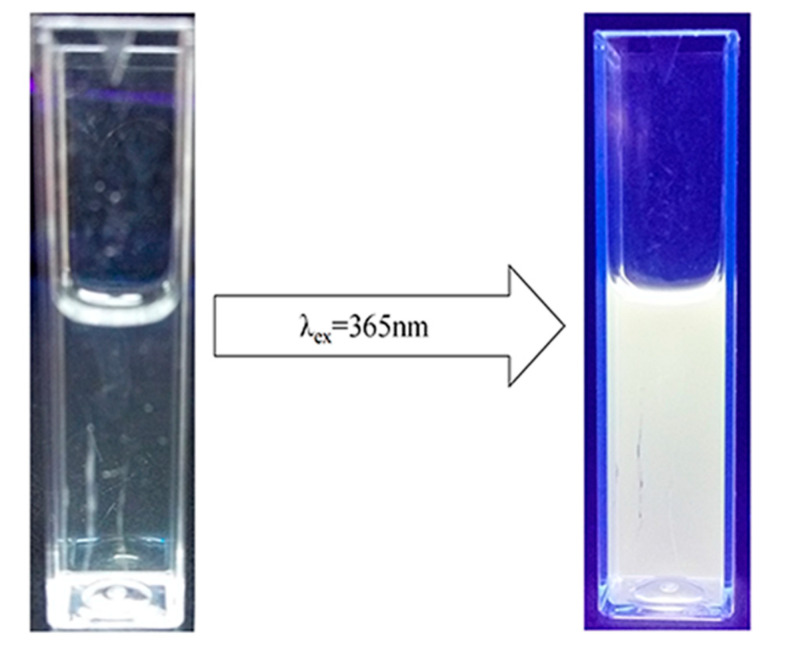
Fluorescence of ZnO quantum dots at λ_ex_ = 365 nm. Left: image of ZnO quantum dots under white light; right: image of ZnO quantum dots under 365-nm ultraviolet light.

ZnO/PVP QDs were synthesized using an improved sol–gel method. To verify that the solution contained ZnO QDs, the solution was subjected to irradiation under UV light at a wavelength of 365 nm. The solution turned from clear and transparent to emit yellow fluorescence. Based on the yellow fluorescence, the fluorescence wavelength was approximately 500–600 nm. The fluorescence spectrum of this solution was red-shifted (Figure 1). The Stokes shift of ZnO occurs only at the quantum level. Upon comparison with the absorption spectrum, it was found that the ZnO QDs were synthesized successfully.

### 2.2. Characterization of ZnO/PVP QDs

**Figure 2:**
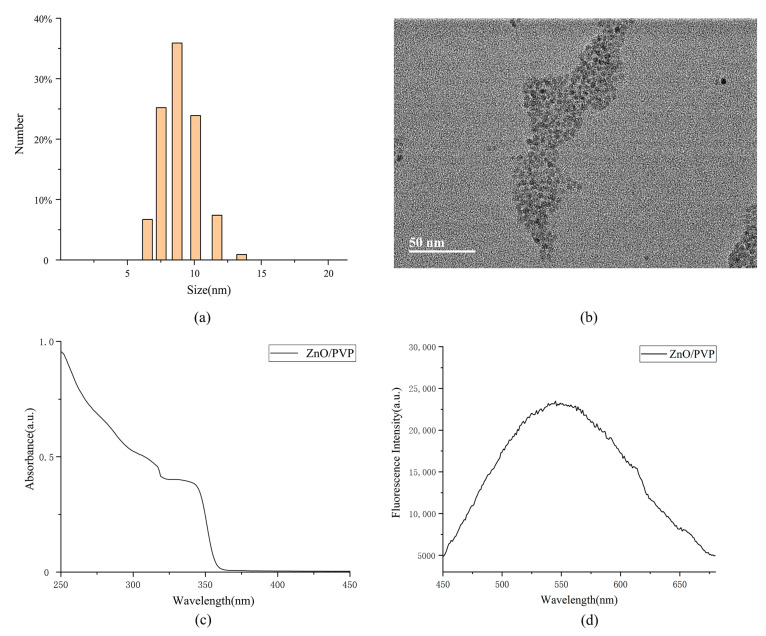
Characterizations of the ZnO/PVP QDs. (**a**) Particle sizes of the ZnO/PVP QDs as measured by DLS, (**b**) TEM image of the ZnO/PVP QDs, (**c**) UV-Vis absorption spectra of the ZnO/PVP QDs, and (**d**) fluorescence spectra of the ZnO/PVP QDs.

The ZnO QD particles were approximately 10 nm, as measured by dynamic light scattering (DLS), indicating that the synthesis of ultrasmall ZnO QDs was successful (Figure 2a). The zeta potential was −3.6 mV on the surfaces of the ZnO/PVP QDs (Figure S1b). Transmission electron microscopy (TEM) showed that the ZnO/PVP particles were well-dispersed in the ethanol solution, with fine, round, and granular characteristics. This also supports that ZnO QDs modified with PVP were more dispersive and less prone to agglomeration. The manual measurement of 50 units of ZnO particles showed that ZnO/PVP possessed appreciable homogeneity at sizes of ~5 nm. The particle sizes appeared larger by DLS compared to those by TEM because of hydration. Upon enlargement of the TEM image, the lattice fringes of ZnO were observed, indicating that the ZnO nanocrystals were successfully synthesized (Figure 2b). The X-ray diffraction pattern of the 2θ values of the ZnO/PVP QDs was obtained in the range of 20–80° (Figure S1a). The peaks were centered at 2θ = 31.75°, 34.28°, 36.15°, 47.58°, 56.51°, 62.86°, and 66.75°, indexing the (100), (002), (101), (102), (110), (103), and (112) diffraction planes, respectively, of ZnO/PVP. The ZnO/PVP QDs belong to the hexagonal wurtzite structure (JCPDS PDF #36-1451). The average crystallite size of the ZnO/PVP QDs (8 nm) was calculated using the Debye-Scherrer formula: D = 0.89λ/βcosθ, where λ is the X-ray wavelength, λ = 0.154184 nm, β is the peak width at half-maximum, and θ is the Bragg diffraction angle. This result is consistent with those of TEM and DLS. UV-Vis and fluorescence spectroscopy analyses were also performed using ZnO/PVP. The fluorescence spectra of ZnO/PVP showed strong absorption in the 250–360-nm UV light band. The spectral absorption of the ZnO QDs was enhanced with decreasing wavelengths, further demonstrating the excellent UV absorption of the ZnO QDs. In addition, the ZnO/PVP spectrum showed a plateau at 320–350 nm. This was one of the unique absorption peaks of the ZnO QDs as synthesized using the sol–gel method, confirming that the ZnO QDs particles were well-distributed (Figure 2c). The fluorescence spectra of ZnO/PVP displayed a broad peak at 500–650 nm. This conforms with the luminescence rule of the ZnO QDs and the yellow fluorescence of the ZnO QDs visible to the naked eye under ultraviolet light irradiation (Figure 1). The fluorescence intensity of ZnO/PVP reached 20,000 a.u. at 560 nm (Figure 2d); emissions at 560 nm can be used for fluorescent labeling. For example, when ZnO/PVP is loaded with anticancer drugs into tumor cells, the location of the drugs is determined by fluorescence, which is important when investigating treatment mechanisms.

### 2.3. Quantum Yield of the ZnO/PVP QDs

Quantum yield refers to the utilization of quantum principles in photochemical reactions; it is defined as the ratio of the number of photons emitted to the number of photons absorbed. This is measured by obtaining the ratio of the fluorescence intensity to the intensity of the absorption. It is typically calculated by comparing the quantum yield of a material with that of a reference material in a certain range.

The quantum yield of ZnO/PVP was calculated according to:

(1) $$Y_{u}=Y_{s}\times \frac{F_{u}}{F_{s}}\times \frac{A_{s}}{A_{u}}$$

In the formula, *Y_u_* represents the quantum yield of the unknown sample, *Y_s_* represents the fluorescence quantum yield of the reference materials, *F_u_* represents the integral fluorescence intensity of the dilute solution of the sample to be measured, *F_s_* represents the integral fluorescence intensity of the dilute solution of the reference materials, and *A_u_* and *A_s_* represent the maximum absorbance values of the sample and reference at the excitation wavelength, respectively. The reference material used in this experiment was rhodamine B, which has a quantum yield of 67% at an absorption wavelength of 365 nm.

The quantum yield of the ZnO/PVP synthesized in this study was 8.7%. This value agrees with the law of quantum yield, indicating an excellent quantum yield of the ZnO QDs. van Dijken et al. [60] showed that smaller fresh ZnO NPs had higher quantum yields. Modified PVP40 on the surface of ZnO protects the acetate groups, prevents tumor growth and aggregation, and maintains optimal luminescent properties.

### 2.4. In Vitro Stability of ZnO/PVP QDs

The ZnO/PVP QD solution was stored in a tin-coated centrifuge tube for 2 weeks. The results of the DLS and TEM showed no significant changes in the ZnO/PVP QDs, and their sizes did not increase significantly. The UV-Vis absorption and fluorescence spectra showed decreased absorption to various degrees. This may be attributed to lower agglomeration of the ZnO QDs, partial restoration of defects on their surfaces, and reduction of the surface area, which may affect their optical properties.

**Figure 3:**
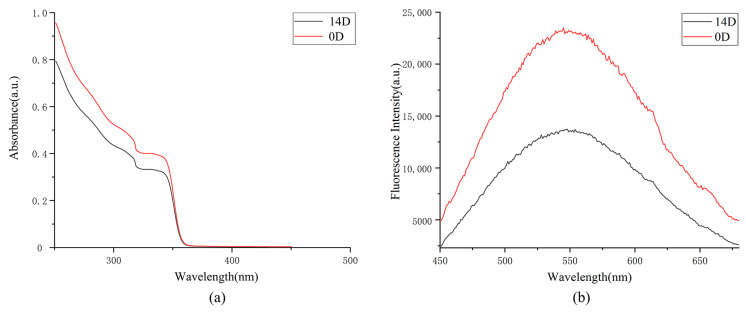
Absorption and fluorescence spectra of the ZnO/PVP QDs stored in an ethanol solution for two weeks. (**a**) absorption spectra of the ZnO/PVP QDs and (**b**) fluorescence spectra of the ZnO/PVP QDs.

Shi et al. [61] reported that unmodified ZnO QD solutions are turbid and agglomerate only after 3 days, suggesting that ZnO QDs without surface modifications are unstable. In this study, the ethanol solution of the ZnO QDs was stored for 14 days, causing the solution to become clear and transparent and allowing fluorescence to be observed under a UV light. The possibility of extensive agglomeration of the ZnO QDs was low. This showed that the polyethylene glycol modification enhanced the stability of the solution, allowing the ZnO QDs to remain stable after 14 days (Figure 3).

### 2.5. Photoluminescent Properties of the ZnO/PVP QDs

**Figure 4:**
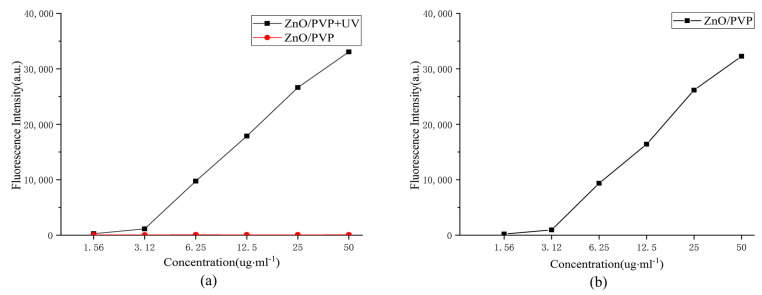
Fluorescence intensities of the ZnO/PVP QDs. (**a**) Fluorescence intensities of the ZnO/PVP QDs and (**b**) fluorescence intensities of the ZnO/PVP QDs at 525 nm.

The photoluminescent properties of the ZnO QDs at different concentrations were determined using a fluorescence microplate reader. The fluorescence intensities of the ZnO QDs increased with the increasing concentrations, indicating that the fluorescence intensities of the ZnO QDs were concentration-dependent. The fluorescence intensity of the 50-μg/mL ZnO/PVP solution was 32,000 a.u., which is consistent with the trend observed for the fluorescence spectra of the ZnO QDs. The change in fluorescence intensities of the ZnO QDs gradually decreased, which may be associated with photon-quenching caused by the increasing concentrations. This also showed that the PVP40 modification exerted no remarkable effect on the optical properties of ZnO (Figure 4a).

### 2.6. In Vitro ROS Production

The ROS levels were determined using 2′,7′-dichlorodihydrofluorescein diacetate (DCFH-DA), which is oxidized by ROS to produce highly fluorescent DCF. The fluorescence of DCF was measured with a fluorescence microplate reader at excitation and emission wavelengths of 488 and 535 nm, respectively. DCHF-DA was added to the ZnO QD solutions at different concentrations, and the fluorescence intensities were measured (Figure 4b). Fluorescence at 525 nm was detected at a concentration of 6.25 μg/mL under UV irradiation, indicating that a ROS was produced at this concentration. The fluorescence intensity of the ZnO/PVP solution at a concentration of 50 μg/mL was 6900 a.u. However, fluorescence at 525 nm was not detected when no UV irradiation was applied.

### 2.7. Cytotoxicity of ZnO/PVP QDs

**Figure 5:**
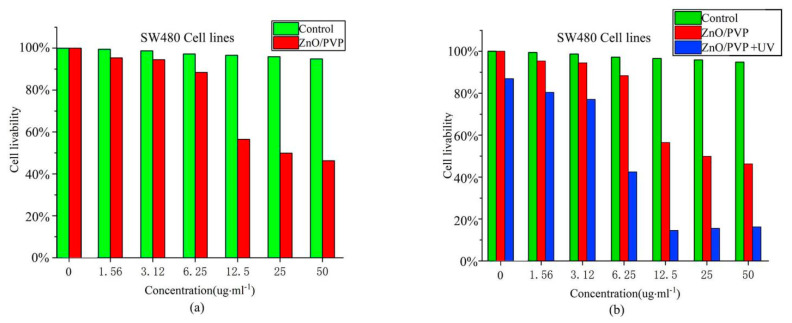
Cell survival rates. (**a**) Cell survival rates of the ZnO/PVP QDs at different concentrations. (**b**) Cell survival rates of the ZnO/PVP and ZnO/PVP+UV groups at different concentrations.

SW480 cells were incubated with different concentrations of ZnO/PVP QDs, and their survival rates were determined in a 3-(4,5-dimethylthiazol-2-yl)-2,5-diphenyltetrazolium bromide (MTT) assay. HEK293T cells were treated in a manner for comparison with SW480 cells. The MTT assay was conducted to measure the cellular metabolic activity as an indicator of cell viability, proliferation, and cytotoxicity. This colorimetric assay was based on the reduction of a yellow tetrazolium salt MTT to purple formazan crystals by metabolically active cells. Viable cells can reduce the MTT reagent, where apoptotic cells cannot. The cell survival rate gradually decreased from a concentration of 12.5 μg/mL and was 54% when the concentration reached 50 μg/mL (Figure 5a). The cytotoxicity of ZnO/PVP may be attributed to the low biocompatibility of PVP40.

### 2.8. Photodynamic Experiment of the ZnO/PVP QDs In Vitro

To evaluate the PDT efficacy of the ZnO/PVP QDs, the viabilities of SW480 and HEK293T cells subjected to UV irradiation were evaluated using an MTT assay. ZnO/PVP showed excellent biocompatibility, with no remarkable changes in cell viabilities under suitable concentrations and without UV treatment. However, ZnO/PVP QDs subjected to UV irradiation showed evident tumor inhibition. Furthermore, the cell mortalities gradually increased with the increasing concentrations, with the cell viability decreasing to 15% at a concentration of 50 μg/mL. Near-complete apoptotic tumor cells were also observed, indicating that ZnO QDs have excellent photodynamic effects in vitro. ZnO/PVP showed evident tumor inhibitory effects at a concentration of 6.25 μg/mL when subjected to UV irradiation. Additionally, the cell survival rate of the group at this concentration was 45% lower than that of the no UV treatment group at the same concentration (Figure 5b). The percentages of apoptotic and necrotic SW480 and HEK-293T cells were analyzed by flow cytometry (Figure S3). The number of necrotic cells decreased after treatment with 25-µg/mL ZnO QDs in both cell types.

The half-maximal inhibitory concentration of ZnO/PVP under UV irradiation was estimated as 21.688 μg/mL. This result is consistent with the trend in ROS produced by ZnO QDs in vitro. Therefore, tumor inhibition of the ZnO QDs may be caused by the excitation of ZnO/PVP by UV light. This causes the generation of electrons and holes that can be transferred to the surface, subsequently generating ROS and inducing tumor cell apoptosis. The beneficial effects observed in the photodynamic in vitro study of the ZnO/PVP QDs motivated us to study their application in PDT in vivo.

### 2.9. Photodynamic Experiment of ZnO/PVP QDs In Vivo

**Figure 6:**
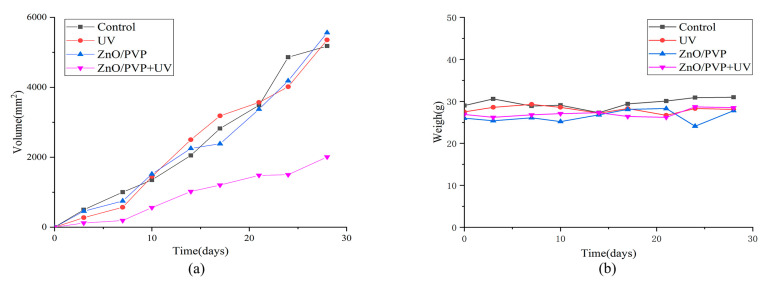
Influence of the treatment on the tumor volumes and weight of tumor-bearing mice treated with PDT. (**a**) Influence of the treatment on tumor volumes of groups of tumor-bearing mice treated with PDT. (**b**) Influence on the tumor weights of groups of tumor-bearing mice treated with PDT.

**Figure 7:**
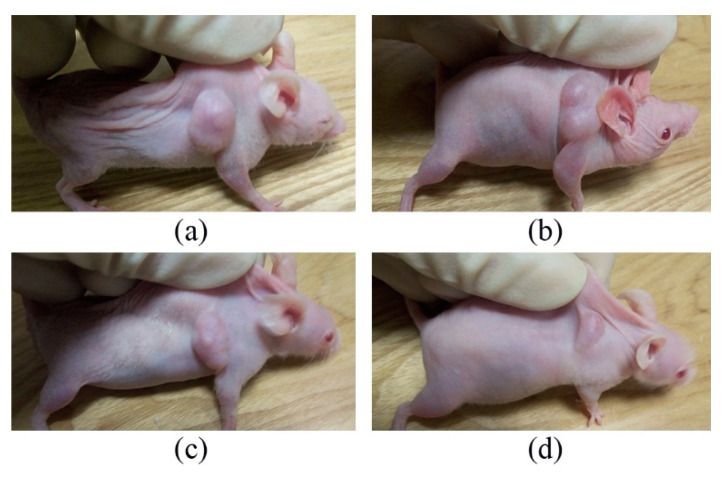
Changes in the tumors of tumor-bearing mice on day 7. (**a**) Control group, (**b**) UV group, (**c**) ZnO/PVP group, and (**d**) ZnO/PVP+UV group.

After dividing tumor-bearing nude mice into four groups, the tumors were measured and weighed every 3 days. The tumor volumes of the control, UV, ZnO/PVP, and ZnO/PVP + UV groups were 5180 ± 759, 5356 ± 795, 5562 ± 480, and 2011 ± 37 mm^3^, respectively, on day 28 (Figure 6). The tumor inhibition rate of the ZnO/PVP+UV group was 61.1%, which was significantly higher than that of the control, UV, and ZnO/PVP groups (*p* < 0.05 in all instances). Moreover, the tumor inhibition effects of ZnO/PVP were observed in the early stages. The sizes of the tumors in nude mice were visible to the naked eye after 7 days (Figure 7 and Table 1).

The tumors were dissected and weighed after the mice were euthanized. The weights of the tumors from the control, UV, ZnO/PVP, and ZnO/PVP+UV groups were 2.17 ± 0.07, 2.17 ± 0.12, 2.08 ± 0.08, and 1.78 ± 0.10 g, respectively (Table 2). The tumor weights in the ZnO/PVP+UV group were significantly lower than those in the control, UV, and ZnO/PVP groups (*p* < 0.05 in all instances).

We observed no significant weight changes between each group throughout the treatment, and the weight fluctuations in each group were less than 1 g (Figure 6b). These results indicate that the injection of the ZnO QDs had no evident toxic side effects on the nude mice, suggesting the good biocompatibility of the ZnO QDs. The basic principle of photodynamic therapy is that the use of a photosensitizer exposed to a certain wavelength of light will be excited to produce ROS, which can kill cancer cells. The tumor inhibition of ZnO QDs may be caused by the excitation of ZnO/PVP by UV light. The results of the in vivo experiments also proved the photodynamic effect on SW480 tumor cells.

Our results demonstrated that ZnO has strong photodynamic therapeutic effects. As the main mechanism of PDT in cancer treatment, ZnO QDs irradiated with ultraviolet light can generate ROS, such as superoxide anion, hydroxyl radical, hydrogen peroxide, singlet oxygen, and peroxyl radicals. These species disrupt the cell membrane integrity and damage lysosomes, mitochondria, proteins, intranuclear macromolecules, and other important physiological functions to finally cause cell apoptosis, thus effectively killing tumor cells in tumor treatments (Figure S4).

Despite the promising therapeutic potential applications of photodynamic tumor suppression, some limitations remain to be addressed. For example, UV exposure at a higher level may cause skin burns. Although the ZnO QDs showed therapeutic potential, further studies are warranted prior to clinical application.

## 3. Materials and Methods

### 3.1. Reagents and Cell Lines

Zinc acetate dihydrate [Zn(OAc)_2_·2H_2_O] and PVP40 [(C_6_H_9_NO)_n_] were purchased from Sigma-Aldrich (St. Louis, MO, USA). Lithium hydroxide (LiOH), anhydrous ethanol, and cyclohexane were purchased from Sinopharm Chemical Reagent (Shanghai, China). All reagents used in this study were of analytical grade. The human colon cancer cell line SW480 was acquired from the cell bank of the Chinese Academy of Sciences (Shanghai, China). The cell lines were cultured in 90% DMEM/L-15 (Gibco, Grand Island, NY, USA) and supplemented with 10% heat-inactivated fetal bovine serum (Gibco). All cultures were maintained in an incubator at 37°C with 5% CO_2_ in a humidified atmosphere.

### 3.2. Synthesis of the ZnO/PVP QDs

First, 220 mg of zinc acetate dihydrate and 66 mg of PVP40 were dissolved in 10 mL of ethanol. The solutions were heated under reflux at 70 °C with magnetic stirring for 1.5 h to allow the formation of a colorless transparent ZnO precursor solution. Meanwhile, 15 mg of LiOH was added to 6 mL of ethanol and incubated at 50 °C for 20 min with magnetic stirring. Subsequently, 10 mL of ZnO precursor solution was added to the LiOH solution and incubated at 50 °C for 1 h with magnetic stirring. Next, 15 mL of ZnO/PVP QD ethanol solution was added to 30 mL of *n*-hexane, and the solution was left overnight at 25 °C. The obtained solution was then centrifuged (Allegra^®^ X-30 Centrifuges; Beckman Coulter, Brea, CA, USA) at 2000 rpm for 10 min, and the resulting supernatant was discarded. Finally, 15 mL of anhydrous ethanol was added to dissolve the precipitate, yielding a clear and transparent solution. ZnO/PVP QDs were obtained.

### 3.3. Characterizations of the ZnO/PVP QDs

The morphology and particle size of the ZnO/PVP QDs were characterized by performing TEM (JEM-2100; JEOL, Tokyo, Japan) and DLS (Zetasizer Nano Z; Malvern Panalytical, Malvern, UK), respectively. X-ray diffraction ((Ultima IV; Rigaku, Tokyo, Japan) of the ZnO/PVP QDs was recorded at room temperature at a scan rate of 2.4°/min from 20° to 80°. The optical properties of the ZnO/PVP QDs were determined using both a UV-Vis spectrophotometer (LAMBDA 365; PerkinElmer, Waltham, MA, USA) and fluorescence spectrophotometer (FL8500; PerkinElmer).

### 3.4. Quantum Yield of ZnO/PVP QDs

To measure the quantum yield of the obtained ZnO/PVP QDs, we used a standard fluorescent dye (rhodamine B) with a known quantum yield of 67% at 365 nm. ZnO/PVP and rhodamine B were each diluted with ethanol. The fluorescence spectrometer was used at excitation and emission wavelengths of 365 and 450–680 nm, respectively. The slits, including the excitation and emission, were set to 3 nm. The solutions of ZnO/PVP and rhodamine B were measured at the same excitation wavelengths and slit widths. The absorption spectra were recorded at this wavelength.

### 3.5. In Vitro Stability Study of the ZnO/PVP QDs

The color, DLS, and wavelength of the ZnO/PVP QD ethanol solution were observed after storage in a tin-coated centrifuge tube for 2 weeks.

### 3.6. Fluorescence Intensities of the ZnO/PVP QDs

Specific volumes (100 µL) of different concentrations of ZnO/PVP solution (1.56, 3.125, 6.25, 12.5, 25, and 50 µg/mL) were pipetted into the wells of a 96-well plate. The fluorescence intensities were measured using a fluorescence microplate reader (Fluoroskan FL; Thermo Scientific, Waltham, MA, USA) (excitation wavelength: 365 nm, emission wavelength: 570 nm) to obtain the fluorescence intensities at each concentration of the ZnO QDs.

### 3.7. Measurement of ROS Levels

The ROS levels were measured using dissolved DCFH-DA, a fluorescent dye that enables the visualization of ROS. The DCFH-DA was dissolved in dimethyl sulfoxide to obtain a 48-µg/mL DCFH-DA solution. Specific volumes (100 µL) of different concentrations of ZnO/PVP solution (1.56, 3.125, 6.25, 12.5, 25, and 50 µg/mL) were pipetted into the wells of a 96-well plate, and 10 µL of DCFH-DA (48 µg/mL) was added to each well. UV light at a wavelength of 365 nm (the source was situated 5 cm above the plate) was used to irradiate the 96-well plate for 5 and 10 min. ROS detection was performed via a fluorescence quantitative analysis using a fluorescence microplate reader (Fluoroskan, FL, USA).

### 3.8. Cytotoxicity Analysis

SW480 cells in the logarithmic growth phase were digested with 0.25% pancreatin, evenly seeded into a 96-well plate (1 × 10^4^ cells/mL), and cultured for 24 h. The prepared ZnO/PVP solutions were divided into six groups and diluted to concentrations of 1.56, 3.125, 6.25, 12.5, 25, and 50 μg/mL using the cell culture medium. The solutions in each group were pipetted into five wells, and a blank control group consisting of only the culture medium was prepared. HEK293T cells were treated in the same manner. The MTT method was used to determine the cell viability.

### 3.9. In Vitro Photodynamic Study

SW480 cells in the logarithmic growth phase were digested with 0.25% pancreatin, evenly seeded into the wells of a 96-well plate (1 × 10^4^ cells/mL), and cultured for 12 h. The prepared ZnO/PVP solutions were divided into six groups and diluted to concentrations of 1.56, 3.125, 6.25, 12.5, 25, and 50 μg/mL using a cell culture medium. The solutions in each group were pipetted into five wells, and a blank control group consisting of only the culture medium was prepared. HEK293T cells were treated in the same manner. The cells were irradiated for 10 min using UV light at a wavelength of 365 nm and then incubated for 48 h at 25 °C. Cell viability was determined using the MTT method.

### 3.10. Experimental Animals

All animal experiments were performed in accordance with the guidelines of the Institutional Animal Care and Use Committee of General Hospital of Chinese People’s Armed Police Forces, which also approved the research procedures (Permit Number 2011-0039). The surgeries were performed using isoflurane gas anesthesia (3% isoflurane–air mixture), and all efforts were made to minimize suffering.

Female nude mice (aged 7 to 8 weeks old) were purchased from the Laboratory Animal Center of the Chinese Academy of Medical Sciences (Beijing, China) and housed under specific pathogen-free conditions at 25 °C with a relative humidity of 50%. All mice were fed sterilized pellets and allowed access to water ad libitum under a 12-h light and dark cycle. The whole-body weights and tumor volumes of the mice were monitored daily. Mice that lost >20% of their initial body weights were euthanized by carbon dioxide asphyxiation, and the experiment was terminated. No mice died prior to application of the humane endpoint.

Approximately 2 × 10^6^ SW480 cells in 50 μL were implanted subcutaneously in the left underarm of each mouse to enable the formation of solid tumors. The mice were used for the experiments once the tumors reached a diameter of 0.5 cm.

### 3.11. In Vivo Photodynamic Study

The tumor-bearing mice were divided into the following four groups: control, UV, ZnO/PVP, and ZnO/PVP+UV groups. After 48 h, each group was again treated with UV, ZnO/PVP, and ZnO/PVP+UV separately. The body weight and tumor volume of each nude mouse were measured daily.

The tumor volume was calculated according to:

*V_t_* = *W_t_*^2^ × *L_t_*/2 (2)

*V_t_* represents the volume of the tumor, *W_t_* represents the width of the tumor, and *L_t_* represents the length of the tumor. The tumor inhibition rate was defined according to:

*R_ti_* = 1 − *V_e_*/*V_c_* × 100% (3)

*R_ti_* represents the tumor inhibition rate, *V_e_* represents the tumor volume of the experimental group, and *V_c_* represents the tumor volume of the control group.

The mice were euthanized using carbon dioxide, and their tumors were dissected and weighed at the end of the experiment.

### 3.12. Statistical Analysis

Statistical significance was determined by one-way analysis of variance, followed by the Newman–Keuls test. All statistical analyses were performed using SPSS 16.0 software (SPSS, Inc., Chicago, IL, USA). *p* < 0.05 was considered to indicate statistically significant results.

## 4. Conclusions

We synthesized ZnO/PVP QDs via an improved sol–gel method and demonstrated their stability and optical properties. Furthermore, we investigated the photoluminescent properties of the ZnO/PVP QDs. PVP40 accumulated at the defect sites on the ZnO surfaces, a mechanism that may be related to photon-quenching at the increasing concentrations. Our in vitro and in vivo studies demonstrated that ZnO/PVP considerably enhanced the PDT efficacy. Therefore, further research of ZnO/PVP bioimaging in live animals is warranted. Based on these results, the ZnO/PVP QDs have potential in the field of photodynamic tumor suppression. We analyzed the mechanism of the photocatalytic killing of cancer, which provided a theoretical foundation for applying ZnO in tumor PDT treatments and a basis for the further synthesis, selection, and modification of new nanomaterials.

## Figures and Tables

**Table 1:** Effects of UV ultraviolet irradiation and non-irradiation on the tumor volumes in mice treated with ZnO QDs.

Group	Number of Nude Mice	Tumor Volume of Nude Mice (mm^3^)				Tumor Inhibition Rate (%)
		M1	M2	M3	$\overset{—}{\chi }$ ± S	
Control group	3	5292	4200	6050	5180 ± 759	–
UV group	3	4400	5320	6348	5356 ± 795	−3.3
ZnO/PVP group	3	5000	4630.5	4400	4543 ± 102	12.3
ZnO/PVP+UV group	3	2025	2048	1960	2011 ± 37	61.1

M1–3: mice 1–3.

**Table 2:** Effects of UV irradiation and non-irradiation on the tumor weights in mice treated with ZnO QDs.

Group	Number of Nude Mice	Tumor Volume of Nude Mice (mm^3^)			
		M1	M2	M3	$\overset{—}{\chi }$ ± S
Control group	3	2.27	2.16	2.08	2.17 ± 0.07
UV group	3	2.3	2.22	2.01	2.17 ± 0.12
ZnO/PVP group	3	2.14	2.13	2.24	2.17 ± 0.04
ZnO/PVP+UV group	3	1.5	1.43	1.52	1.48 ± 0.03

M1–3: mice 1–3.

## Data Availability

The data presented in this study are available in this article.

## Supplementary Materials

The following are available online at https://www.mdpi.com/article/10.3390/ijms22158106/s1.
