# Supplementary material for: Synthesis and Characterization of Polyvinylpyrrolidone-Modified ZnO Quantum Dots and Their In Vitro Photodynamic Tumor Suppressive Action
Source: Int J Mol Sci. 2021 Jul 28;22(15):8106. doi: 10.3390/ijms22158106 (PMC8347431; doi:10.3390/ijms22158106)
Supplement: Supplementary file 1 [file ijms-22-08106-s001.zip › ijms-1301794-supplementary.pdf]

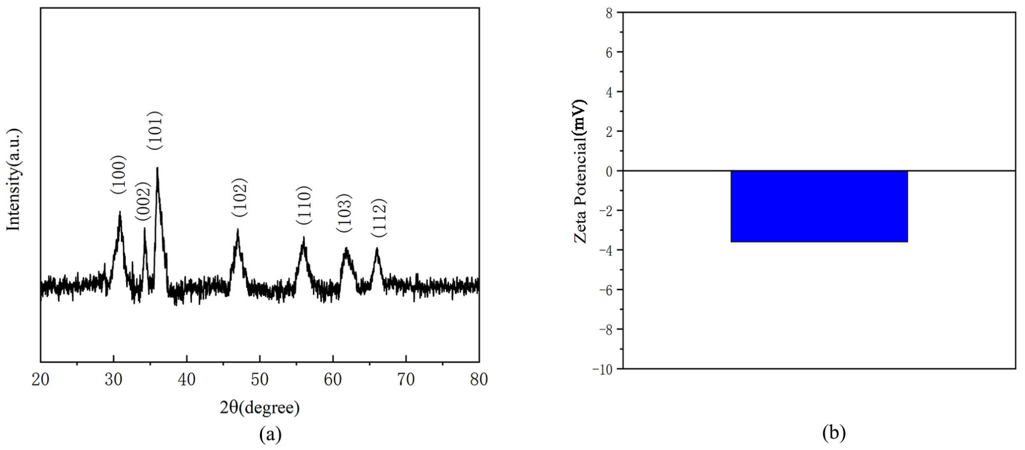

Figure S1. (a) The XRD spectrum of the ZnO/PVP QDs; (b) Zeta potential of the ZnO/PVP QDs;

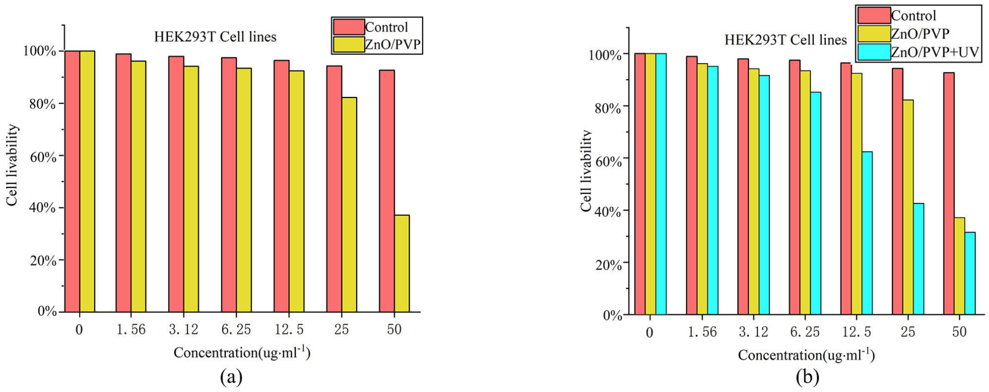

Figure S2. Cell survival rates of HEK293T cell lines. (a) Cell survival rates of the ZnO/PVP QDs at different concentrations; (b) Cell survival rates of the ZnO/PVP and ZnO/PVP+UV groups at different concentrations.

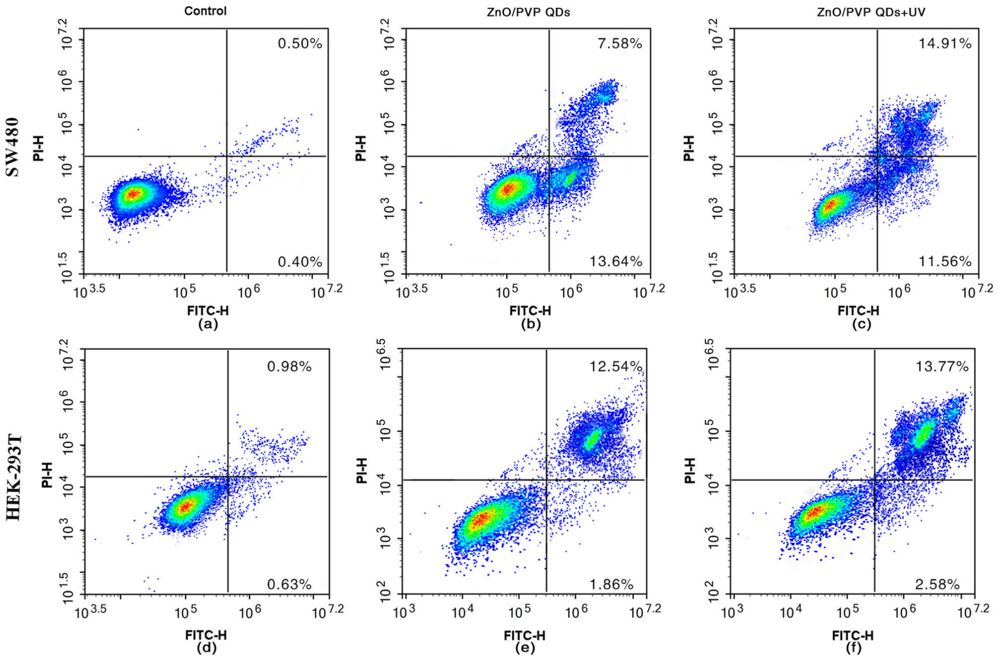

Figure S3. Annexin V/PI staining combined with flow cytometry was used to determine apoptosis of SW480 and HEK-293T cells line which induced by ZnO/PVP QDs at the concentration of 25μg/ml.

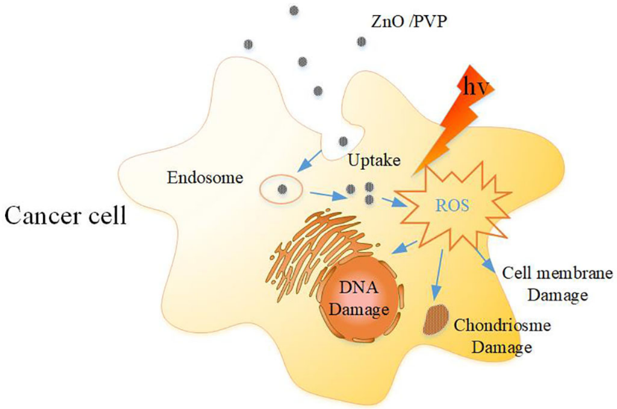

Figure S4. Schematic illustration of the mechanism of photodynamic tumor suppressive action of ZnO/PVP QDs
